# Nano-cellulose biopolymer based nano-biofilm biomaterial using plant biomass: An innovative plant biomaterial dataset

**DOI:** 10.1016/j.dib.2018.02.053

**Published:** 2018-02-26

**Authors:** A.B.M. Sharif hossain, Musamma M. Uddin, Mohammad Fawzi, Vajid N. Veettil

**Affiliations:** a Program of Biotechnology, Department of Biology, Faculty of Science, University of Hail, Hail 2440, KSA; b Biotechnology Program, Institute of Biological Science, Faculty of Science, University of Malaya, Kuala Lumpur, Malaysia

**Keywords:** Nanocellulose, Nanobiofilm, Nanobioplastic, Biodegradable, Corn leaf

## Abstract

The nano-cellulose derived nano-biofilm keeps a magnificent role in medical, biomedical, bioengineering and pharmaceutical industries. Plant biomaterial is naturally organic and biodegradable. This study has been highlighted as one of the strategy introducing biomass based nano-bioplastic (nanobiofilm) to solve dependency on petroleum and environment pollution because of non-degradable plastic. The data study was carried out to investigate the nano-biopolymer (nanocellulose) based nano-biofilm data from corn leaf biomass coming after bioprocess technology without chemicals. Corn leaf biomass was used to produce biodegradable nano-bioplastic for medical and biomedical and other industrial uses. Data on water absorption, odor, pH, cellulose content, shape and firmness, color coating and tensile strength test have been exhibited under standardization of ASTM (American standard for testing and materials). Moreover, the chemical elements of nanobiofilm like K^+^, CO_3_^−−^, Cl^−^, Na^+^ showed standard data using the EN (166).

**Specification table**

**Table:** 

Subject area	Biological chemistry, Biochemistry
More specific subject area	Nanocellulose based nanobiofilm bioplastic from plant biomass
Type of data	Physicochemical, mechanical (Table and Figure)
How data were acquired	SEM, pH meter, spectrophotometer, Tensile test, absorption test, burning test, bore, shape and size test, chemical test by ASTM and EN standard.
Data format	Row data were collected and analyzed
Experimental factors	Single factor
Experimental features	Three replicates were used in the experiment as Randomized Complete Design (CRD). The sample was selected randomly from the different lots.
Data source location	Kuala Lumpur, Malaysia and Hail, KSA
Data accessibility	This is an innovative data, not yet published elsewhere.

**Value of the data**

1. Data have been highlighted bearing innovative information on nano-biofilm or other definite biomaterials for medical, biomedical and bioengineering industries from corn leaf biomass.
2. Data exhibited a outstanding and an innovative research. Data would be a valuable to the related researcher and academician, on nano-biofilm production using plant biomass as plant biomaterial.
3. Data investigated the appropriate quality of nano-cellulose based nano-biofilm plant biomaterial production using agro-biomass according to the ASTM (American standard for testing and materials) and EN (European Norms) standardization.
4. Data can be explored for the future studies in the related research community all over the world.

## 1 Data

**Fig. 1:**
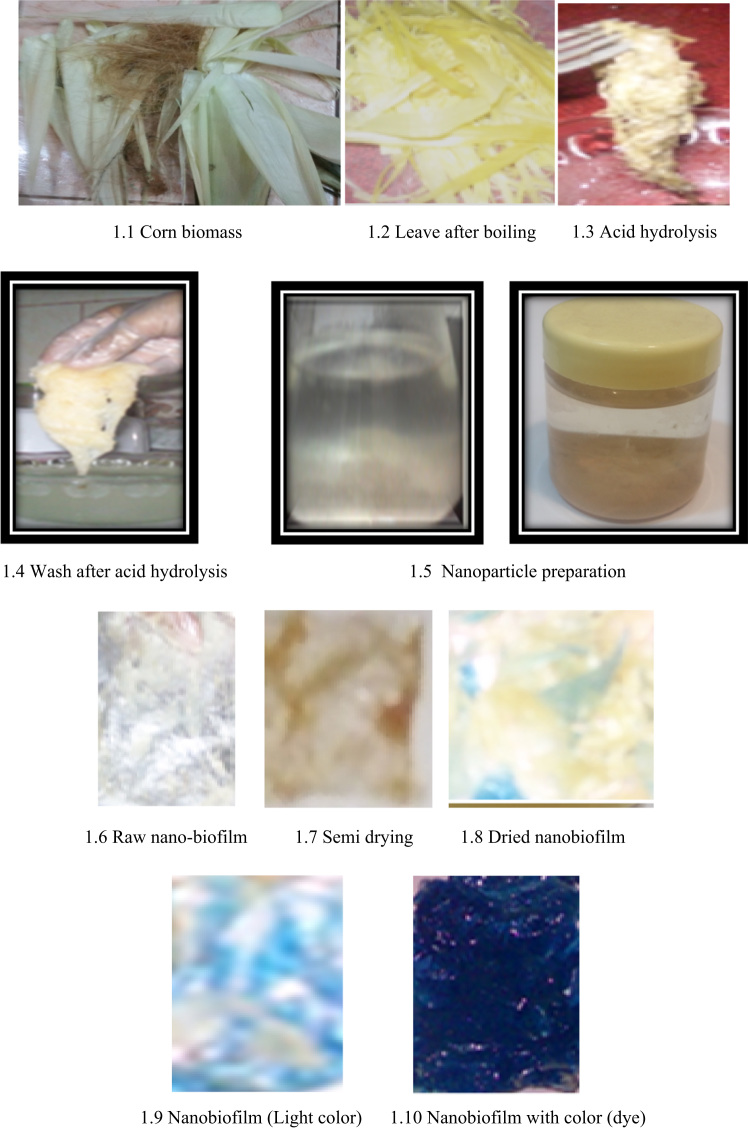
Nanobiofilm production procedure from corn leaf samples.

**Fig. 2:**
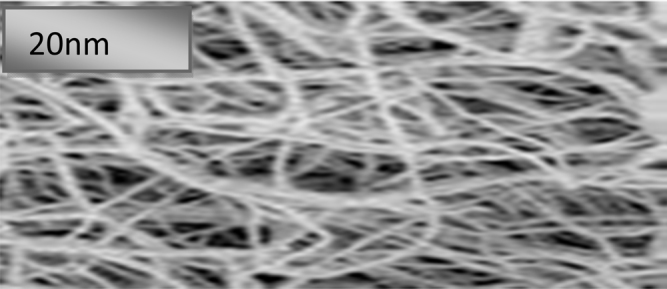
Nanocellulose size measurement.

Data show the nano-biofilm production procedure derived from nanocellulose based corn leaf biomass (Fig. 1). Data observe the nanosized biofilm as nanocellulose detected by Transmission Electron Microscopy (TEM) (Fig. 2) (Table 1). In Table 2, data exhibit the negligible water percent absorbed by nanobiomaterial based nano-biofilm. Moreover, data of the odor, color of flame and speed of burning represented by burning test were no odor, yellow–orange flame and slow speed of burning respectively which were under the standardization of burning test (Table 3). In addition, data describe on the color dying time for drying at different hours (Table 4). Table 5 shows the tensile strength and tensile modulus for the nano-bioplastic derived nanobiofilm. Data mentioned in Table 6, show the positive shape and size test for the nano-biofilm. In Table 7, data observe the value on pH and cellulose. In addition, chemical elements test data from nanobiofilm samples like K^+^, CO_3_^−−^, Cl^−^, Na^+^ were measured and represented data using the EN (166)) standardization (Table 8).

**Table 1:** Measurement of nanocellulose by Transmission Electron Microscopy (TEM).

Materials	Nanocellulose size
Corn leaves sample as raw material of Nano-biofilm	20 nm
Nanoparticle size	1–100 nm

**Table 2:** Determination of water absorption by ASTM D570.

Materials	Water absorption	ASTM D570
		Water absorption
Nano-biofilm	0.03%	Water absorption by ASTM is 0–0.16%.
Synthetic film	0–0.16%

**Table 3:** Odor test according to the ASTM D3801.

Materials	Odor	Color of flame	Speed of burning	Spark or not
Nano-biofilm	No odor	Yellow–orange	Slow	Spark
Synthetic film				
By ASTM D3801	Low odor	Yellow–orange	Slow	Spark

**Table 4:** Color coating dye was used as the mode of application by ASTM B 117.

Materials	Coating dye test	ASTM B117
	(Drying time)	Maximum 2 h
Nano-biofilm	1.3 h	Max. 2 h
Synthetic film	2 h (max).	
By ASTM B 117

**Table 5:** Determination of tensile test by using ASTM by ASTM D5083.

Materials	Tensile strength (MPa/kg.m3)	Tensile Modulus (GPa)
Nano-biofilm	74.0	1.1
Synthetic film		
By ASTM D5083	70–230 (ASTM)	1.0–3.0 (ASTM)

**Table 6:** Firmness test represented by bore and crack test.

	Shape, size and Bore and test	
Materials	Firmness test	Firmness test Shape and size
	Bore test ASTM D2925	by ASTM D500 (Resistant Character)
Nanobiofilm	No bore symptom	No swell or shrink
Synthetic film	No bore symptom	No swell or shrink

**Table 7:** pH and cellulose determination from corn waste biomass sample.

Test	pH	Cellulose
Corn samples	7.3 ± 0.01	65.5% ± 0.05
Plastic film sample	Alkaline ≥ 7	(It is zero if from gas or oil sample, if from cellulose sample it is up to 70%)

Mean±SE (standard Error, *n* = 3).

**Table 8:** Chemical element determination nanocellulose sample.

Chemical Element	Corn sample based Biofilm (PPM)	Synthetic Plastic film By EN (European Standard EN166.)
K^+^	9.7 ± 0.5	10
Na^+^	4.2 ± 0.2	5
Cl^−^	0.55 ± 0.01	2
CO_3_^−−^	139.1 ± 1.1	5–440

Mean ± SE (standard Error, *n* = 3).

## 2 Experimental design, materials and methods

### 2.1 Sample collection and preparation

Five kg corn stalk new leaves were collected from the farmers field, Kuala Lumpur Malaysia and Hail regional area, KSA. Leaves were randomly chosen from both area and removed from corn stalk and washed to clean. Washed leaves were sliced by scissors and boiled (Fig. 1). Then it was blended by blender. After blending it was again ground for fine mixing by motor and pestle and put it to the beaker.

### 2.2 pH determination

The pH was determined by using Horiba Scientific pH meter, Japan.

### 2.3 Cellulose determination

The quantitative determination of cellulose content from the corn sample was made by anthrone reagent [1]. A standard curve was drawn by measuring the absorbance of known concentration of cellulose solutions at 620 nm. Anthrone reagent consisted of acetic nitric reagent, 67% sulfuric acid, 10 ml anthrone solution. The tubes were then kept the boiling water bath for 16 min. The mixture (centrifuged sample 0.2 g/10 ml and chemical mixture) was then cooled in ice bath for 2–3 min and made it normal at room temperature. To measure cellulose content, 3 ml of unknown cellulose solution was filled into a test tube, followed by addition of 3 ml of anthrone reagent and its absorbance at 620 nm was measured.

### 2.4 Samples pyrolysis

Blended and ground sample was heated at 150 °C in pressure cooker for 2 hours at 30 psi until the sample become liquid paste. After heating the liquid fiber samples were cooled down. A 0.8% (w/v) sodium chlorite (NaClO_2_) solution and acetic acid were added to acidify the NaClO_2_ solution until the pH reached 4.5. The fibers were boiled in NaClO_2_ solution for 3 h at 70–80 °C whereby the ratio of fiber to NaClO_2_ solution was set to 1: 30. The bleaching process was repeated for five times until fiber became white and then filtered. After being filtered, the residue was washed for several times with distilled water and dried in air. The bleached cellulose obtained was heated to 70–80 °C in 5% (w/v) sodium sulfite solution for 2 h. The fibers were filtered, washed, and dried in the air. After being dried, the fibers were treated in 17.5% (w/v) sodium hydroxide (NaOH) solution for 2 h. The residue was washed for several times with distilled water.

### 2.5 Nano-particle preparation by acid hydrolysis

Fiber sample was hydrolyzed (100 ml/50 g sample) by hydrochloric acid (HCl 99% pure) to make it micro to nano size particle for 12 h. The water bath (60 °C) was used during the process of hydrolysis occurred. After 12 h the samples were separated by separation funnel and washed by distilled water five times (Fig. 1).

### 2.6 Nanoparticle measurement

Nano particle size was measured by Transmission electron microscopy (TEM) (Fig. 2). TEM images were obtained using a JEM-2100 transmission electron microscope operated at 120 kV. For TEM sample preparation, the nanocellulose particles were deposited on a carbon-coated grid by placing a drop of a very dilute cellulose nanofiber suspension on the grid and then allowed to dry in order to evaporate the liquid.

### 2.7 Plasticizer mixture

Acetic acid 5% (5 ml/100 g cellulose sample), 5 ml/100 g (polyvinyl chloride), and starch powder 20%, and 20% water were added with the 500 g of cellulose (65.5%) samples. Later 10 ml/100 g PVC (polyvinyl chloride) and glycerin (5 ml/100 g) were added with the mixture of cellulose samples and waited for 10 min to mix up well. Then the mixture was heated at 150 °C in the oven for 30 min at 30 psi pressure followed by pyrolysis method as ASTM standard until visual plasticity occurred in the oven for nanobioplastic film material. The raw nanobiofilm were taken it out from the oven and kept it in room temperature at 28 °C for cooling down for 10 min. Then raw nanobiofilm was put in the aluminum foils to make it dry for 30 min. Al last, semidried nano-bioplastic film was oven dried at 80 °C for 2 h. The completely dried nanobioplastic film was used for different tests to investigate the fitness.

### 2.8 Testing fitness

#### 2.8.1 Absorption test (as ASTM D570) [2]

**Fig. 3:**
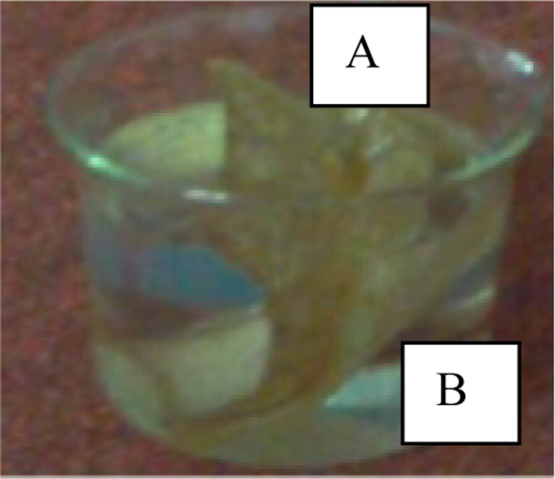
Absorption test. Nanobiofilm (A) in the water cylinder (B).

For the water absorption test, the specimens were dried in an oven for a specified time and temperature and then placed into the desiccator to cool. Immediately upon cooling the specimens were weighed. The material was then emerged in water at agreed upon conditions, often 23 °C for 48 h. Specimens were removed, patted dry with a lint free cloth and weighed (Fig. 3). The diameter of disk was 5 cm and 2 mm thick. Water absorption was calculated.

#### 2.8.2 Odor test

**Fig. 4:**
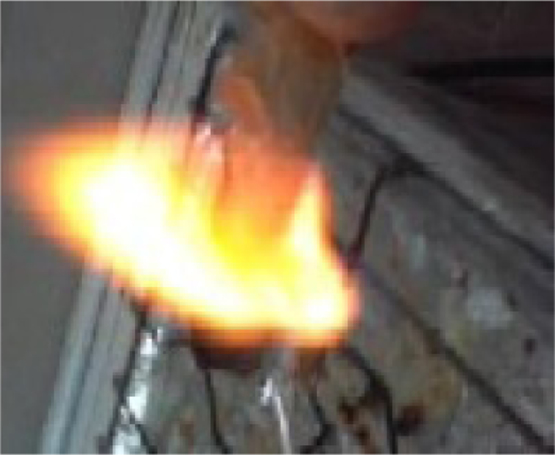
Burning test.

It was burnt by using gas burner. Odor, color of flame, speed of burning and spark were observed by visual observation and compared with the synthetic bumper by ASTM D3801 (Fig. 4).

#### 2.8.3 Tensile/tension test

Tensile test was done by Universal Test Machine for bioPlastics as ASTM D5083 [3].

##### 2.8.3.1 Test procedure

Specimens were placed in the grips of a Universal Test Machine at a specified grip separation and pulled until failure. For ASTM D5083 the test speed was measured by the material specification. The default test speed was 5 mm/min (0.2 in/min), but modulus determination was made at 1.5 mm/min (0.057 in/min). A strain gauge was used to determine the elongation and tensile modulus. Max Load Capacity was 50 kN/m^2^ depending upon the reinforcement and type.

##### 2.8.3.2 Sample size

The standard specimen for ASTM [3] has a constant rectangular cross section, 25 mm (1 in) wide and at least 250 mm (10 mm) long. Thickness can be between 1.5 mm (0.057 in) and 14 mm (0.55 in). Optional tabs can be bonded to the ends of the specimen to prevent gripping damage. Intertek PTL can machine the specimens from larger samples and bond tabs if requested. Tensile Strength (MPa or PSI) was displayed from tensile test.

#### 2.8.4 Color test

Spray coating dye was used as the mode of application. It was attached properly with plastic and dried after 1 h (Fig. 1).

#### 2.8.5 Shape and size test

By the hammer it was continuously beaten for 2 min and pulled on for 5 min. There was no change of its shape and size.

#### 2.8.6 Firmness test: (Bore test)

Bioplastic film was hit by the hammer of 1 kg on the screw set on the biofilm. Hit was completed for 5 min.

#### 2.8.7 Chemical element test

Chemical element like K^+^, CO_3_^−−^, Cl^−^, Na^+^ were tested using different meters. K^+^ and Na^+^ were tested by LAQUA twin K+ meter and LAQUA twin Na+ meter (Horiba, Japan). CO_3_^−^, and Cl^−^ were tested by Photometer PF-3,version A (Macherey-Nagel, Germany). In the case of all chemical elements positive results exhibited and compared to the synthetic plastic in the laboratory using the EN (166) standardization (Fig. 1) [4].

#### 2.8.8 Statistical analysis

Randomized Complete Design (CRD). The sample was selected randomly from the different lots in the experiment. Standard deviation was calculated from the mean of the replicates and Standard error was analyzed from standard deviation using 3 replicates of the samples where necessary (*n* = 3) (*n* = replicate).
