# Supplementary material for: Nano-cellulose biopolymer based nano-biofilm biomaterial using plant biomass: An innovative plant biomaterial dataset
Source: Data Brief. 2018 Feb 26;17:1245–52. doi: 10.1016/j.dib.2018.02.053 (PMC5966518; doi:10.1016/j.dib.2018.02.053)
Supplement: Supplementary file 1 — Supplementary material [file mmc1.docx]

**Conflict of interest**

To

The Editor

Data in Brief

Dear Editor

With due respect, we would like to mention that we do not have any conflict of interest in publishing the original research work in the Data in Brief.

Best Regards

Dr. ABM sharif Hossain

(on behalf of all authors)
